# The impact of COVID‐19 pre‐university education on first‐grade medical students. A performance study of students of a Department of Histology

**DOI:** 10.1002/ase.2551

**Published:** 2025-01-11

**Authors:** José Manuel García, David Sánchez‐Porras, Miguel Etayo‐Escanilla, Paula Ávila‐Fernández, Olimpia Ortiz‐Arrabal, Miguel‐Ángel Martín‐Piedra, Fernando Campos, Óscar‐Darío García‐García, Jesús Chato‐Astrain, Miguel Alaminos

**Affiliations:** ^1^ Tissue Engineering Group, Department of Histology, Medical School University of Granada Granada Spain; ^2^ Instituto de Investigación Biosanitaria ibs.GRANADA Granada Spain

**Keywords:** COVID‐19, medical education, performance

## Abstract

The recent coronavirus disease (COVID‐19) forced pre‐university professionals to modify the educational system. This work aimed to determine the effects of pandemic situation on students' access to medical studies by comparing the performance of medical students. We evaluated the performance of students enrolled in a subject taught in the first semester of the medical curriculum in two pre‐pandemic academic years (PRE), two post‐pandemic years (POST), and an intermediate year (INT) using the results of a final multiple‐choice exam. Consistency analysis among periods was performed using the Cronbach alpha coefficient (*α*), the difficulty index with random effects correction (DI), and the point‐biserial correlation index (PB). The five exams were homogeneous and had similar *α*, DI, and PB difficultness. Performance significantly decreased in POST students compared with PRE students, with a correlation between performance and the academic years (PRE‐POST). A significant decrease in the percentage of correct answers was detected in the academic years, with POST students showing lower results than PRE students, but not in the percentage of questions answered incorrectly. Significantly higher percentages of unanswered questions were found among POST students. These results confirm the negative impact of the POST pre‐university educational system on the performance of students accessing medical school and suggest that POST students could have a higher degree of uncertainty. Specific education programs should be implemented during the first years of the medical curriculum to tailor this effect and increase students' self‐confidence and knowledge, which may be associated with confidence.

## INTRODUCTION

Medical education is a complex process that requires continuous evaluation and adaptation to new situations, which may involve rethinking attitudes and reorienting teaching objectives, with the ultimate goals of improving the students' knowledge and skills.^1^

In this context, the recent COVID‐19 pandemic emergency that forced education authorities and professionals to adapt the teaching systems to the unprecedented new situation was an enormous challenge for students worldwide.^2^ In medical schools, modifications were made at different levels. At the clinical level, the pandemic transformed the clinical rotations of residents and medical students and the training strategies applied to surgery,^3^ radiology,^4^ and mental health,^5^ among other medical specialties. In most cases, direct contact with patients was replaced by a web‐based educational system in which simulation and e‐learning strategies were implemented.^6^, ^7^, ^8^ In undergraduate medical education, most medical schools opted to implement several changes to the medical curriculum or adopt different types of student‐led educational activities.^9^ In general, medical teachers were forced to modify their teaching strategies and the way they conducted classes, adopting online distance learning and virtual teaching instead of traditional in‐person teaching methods.^7^, ^10^ These changes might have substantially altered the teaching‐learning process in medical education, as previously demonstrated.^11^, ^12^, ^13^ However, the impact of the new situation on the development of students enrolled in pre‐university studies who subsequently accessed medical studies at medical schools has not been studied in depth; hence, the possible influence of this educational change on the performance of these students at medical schools requires further research.

In Spain, pre‐university education consists of three distinct levels: 6 years of basic studies (primary education), followed by 4 years of secondary education (ESO) and 2 years of high school (*bachillerato*). The last level of pre‐university education is optional and focuses on preparing students for university studies with specific training methods for students who wish to enroll in health science education at the university level. As for medical school students, high school students were substantially affected by the COVID‐19 pandemic, and numerous adaptations were required at this educational level.^14^ Previous reports have demonstrated that educational disruptions affecting high school are significantly associated with students' decreased academic performance.^15^ Whether this situation affected the performance of students enrolled in medical schools has not yet been adequately studied.

In the present study, we evaluated the academic performance of students enrolled in the first academic year of the medical school in Granada, Spain, corresponding to students who received pre‐pandemic and post‐pandemic high school formations, to determine whether the pandemic might have influenced the performance of medical students enrolled in a subject taught in the first semester of the medical curriculum.

## METHODS

### Sample and data source

In this work, we evaluated the scores obtained by students enrolled in the subject “Cell Biology and Principles of Human Genetics and Cytogenetics” which is taught in the first semester of the first academic year (AC) of the Degree in Medicine at the Medical School of the University of Granada, Spain. These scores correspond to each student's performance on the final global examination taken at the end of the AC, which corresponds to a teaching year. The results corresponding to the last five AC were evaluated for: 2019–2020 (AC19‐20), 2020–2021 (AC20‐21), 2021–2022 (AC21‐22), 2022–2023 (AC22‐23), and 2023–2024 (AC23‐24). The teachers and teaching methods used during these five ACs were the same, and the subject contents did not vary among ACs, with the only difference being that pandemic teaching was performed using online resources only for AC20‐21, whereas face‐to‐face learning was used for the rest of the AC.

This exam included 60 multiple‐choice questions (items) related to Cell Biology and Principles of Human Inheritance taught during the AC. For each item, the students were asked to select the correct option from four possibilities (A, B, C, or D), with only one being correct. Selection of the correct option was scored with 1 point over a total of 60 points, incorrect options were penalized at −0.33 points, whereas unanswered questions did not affect the final score. The final scores were then adapted to a scale ranging from 0 to 10 points by dividing the final result (over 60 points) by 6.

### Consistency analysis

To determine the homogeneity of the results obtained by the students in the different exams, we performed several consistency analyses. First, reliability was assessed for each test by determining the alpha coefficient of Cronbach (*α*). This coefficient was calculated as previously reported^16^: $\alpha =\frac{k}{k-1}\left(1-\frac{\sum_{j=1}^{n}\sigma_{j}^{2}}{\sigma_{x}^{2}}\right)$ where k is the number of items in the test, $\sigma_{x}^{2}$ is the global variance of all values, and $\sum_{j=1}^{n}\sigma_{j}^{2}$ is the sum of variances of the options for each question. The *α* coefficient can range from 0 to 1, and the highest consistency corresponds to the highest values of *α*. The difficulty index with random effects correction (DI) was then calculated for each test question using the following formula: $\mathrm{DI}=\frac{A-\frac{E}{k-1}}{N}$, where *A* is the number of students who gave a correct answer to each question, *E* is the number of students who gave an incorrect answer to the question, *k* is the number of possible answers to each question, and *N* is the total number of students in the study. Questions with DI > 0.5, are considered easy, whereas questions with DI < 0.5, are considered difficult.^16^, ^17^ Finally, the scores assigned by the students to each question were analyzed to determine the point‐biserial correlation index (PB) with the following formula^18^: $\mathrm{PB}=\frac{\mu_{p}-\mu_{q}}{\sigma_{x}}\sqrt{\frac{\mathrm{ID}}{1-\mathrm{ID}}}$, where *μ*_*p*_ is the mean score of the students who correctly answered to each item, *μ*_*q*_ is the mean score of the students who incorrectly answered to the item, *σ*_*x*_ is the global standard deviation of all test scores, and ID is the difficulty index of the item determined as the number of students correctly answering the item versus the total number of students.

### Performance analysis

To determine the performance of students enrolled in each AC, the average and standard deviation values of the scores obtained in the final examination were calculated for AC19‐20, AC20‐21, AC21‐22, AC22‐23, and AC23‐24. Averages and standard deviations were also calculated for the students who received pre‐COVID‐19 pre‐university education (PRE), corresponding to AC19‐20 and AC20‐21, students enrolled in post‐COVID‐19 pre‐university education (POST), corresponding to AC22‐23 and AC23‐24, and the group of students who received a mixed intermediate (INT) pre‐university education system (1 year with the pre‐COVID‐19 system and 1 year with the post‐COVID‐19 system), corresponding to AC21‐22.

### Statistical analysis

All distributions were first analyzed for normality using the Shapiro–Wilk test. The results of this test demonstrated that the criteria for the use of parametric comparison tests were not fulfilled; therefore, non‐parametric tests were used. The Kruskal–Wallis test was used to compare the results of several distributions at the same time (e.g., the results of the five ACs). For post hoc pairwise comparisons of two specific groups (e.g., the results of AC19‐20 vs. AC20‐21), we used the Mann–Whitney test. The correlation between two variables (for example, the AC and students' scores) was carried out using Kendall's tau correlation test, and the time‐related trend of a variable was determined by the *R* ^2^ linear trend of the variable. We compared the scores obtained by all students, and male and female students independently, the percentage of items answered correctly and incorrectly, and the percentage of unanswered items. In addition, a binary logistic regression analysis was performed to predict the association between the dichotomized type of sample (PRE or POST) and students' final scores, percentages of correct and incorrect answers, and percentages of unanswered questions. Statistical comparisons were performed using Real Statistics Resource Pack software (Release 7.2) (Dr. Charles Zaiontz, Purdue University, West Lafayette, IN, USA). To correct for multiple testing, a Bonferroni‐adjusted *p*‐value of <0.001 was considered statistically significant. Although not significant, the values between 0.05 and 0.001 were considered marginally significant.

## RESULTS

### Consistency analysis of the test results

A total of 1246 students were enrolled in these five AC, with an average of 249 ± 16 students per AC (32% men and 68% women).

Analysis of the different exams using Cronbach's alpha coefficient (Table 1) showed a high‐reliability index in all cases, with results ranging from *α* = 0.8833 for AC20‐21 to *α* = 0.9549 for AC19‐20.

**TABLE 1:** Analysis of the consistency of the five exams analyzed in the present study. For each academic year (AC), Cronbach's alpha coefficient (*α*), the difficulty index with random effects correction (DI), and the point‐biserial correlation index (PB) are shown.

	AC19‐20	AC20‐21	AC21‐22	AC22‐23	AC23‐24
Cronbach's alpha coefficient (*α*)	0.9549	0.8833	0.9542	0.9354	0.9539
Difficulty index with random effects correction (DI)	68.33%	56.67%	63.33%	55.00%	56.67%
Point‐Biserial Correlation Index (PB)	96.67%	93.33%	91.67%	95.00%	100%

*Note*: For the DI and PB, the percentage of items showing a value >0.5 is shown. AC19‐20: Academic year 2019–2020, AC20‐21: Academic year 2020–2021, AC21‐22: Academic year 2021–2022, AC22‐23: Academic year 2022–2023, and AC23‐24: Academic year 2023–2024.

When the difficulty of the items contained in each test was analyzed using the difficulty index with random effects correction (Table 1), we found that the percentage of items showing low difficulty (DI > 0.50) was above 50% in all cases. Differences between the different exams were not statistically significant (Fisher's exact test *p* > 0.05 for all comparisons), and the correlation analysis between DI and the AC showed that the association between both variables was not statistically significant (Kendall *p* = 0.2122, *r* = 0.0645).

In addition, point‐biserial correlation index analysis (Table 1) revealed very high values in all cases, with more than 90% of the items showing very good results for this parameter. As in the previous case, non‐significant differences were found when PB was compared among the different exams (Fisher's exact test *p* > 0.05 for all comparisons), and the correlation analysis was not statistically significant (Kendall *p* = 0.5689, *r* = 0.0295).

### Performance of the students in each academic year

**FIGURE 1:**
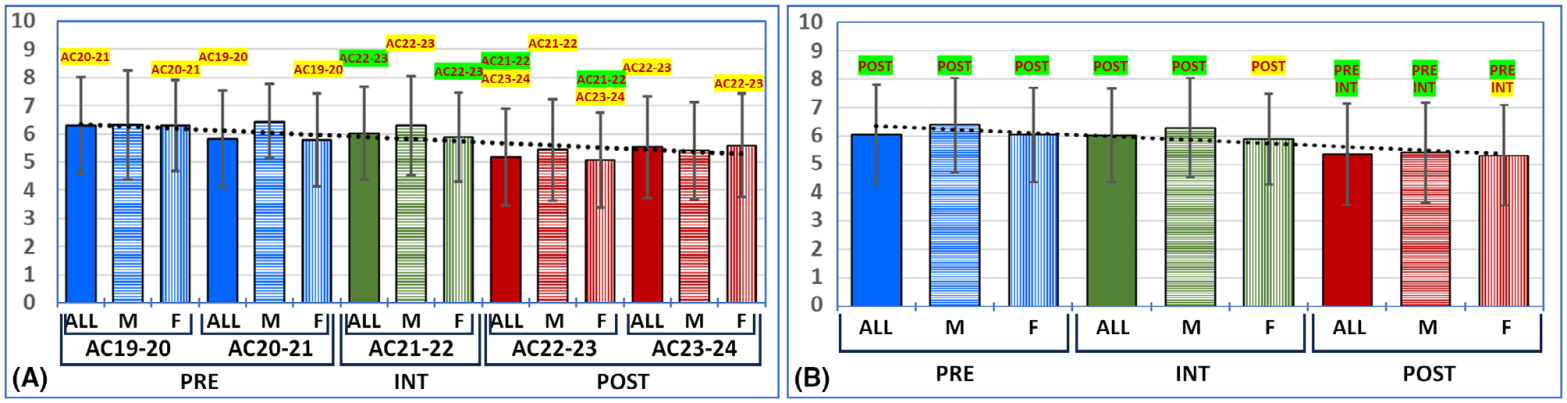
Performance of the students enrolled in each academic year (AC). Results are shown as average values for students enrolled in each AC (panel A) and the PRE, INT, and POST groups (panel B), for the whole group of students (ALL), and for male (M) and female (F) students only. Error bars correspond to standard deviations. PRE: Students who received pre‐COVID‐19 pre‐university education; INT: Students who received a mixed intermediate pre‐university education system; POST: Students enrolled in post‐COVID‐19 pre‐university education. The black dotted lines show the linear trend in panels A and B. The comparison groups showing significant *p* values of <0.001 are shown above each specific group highlighted in green color, whereas comparison groups showing *p* values between 0.05 and 0.001 are shown in yellow.

Analysis of the results obtained by the students enrolled in each study group revealed an average score value ranging from 5.17 ± 1.72 in the academic year AC22‐23 to 6.29 ± 1.71 in AC19‐20 (Figure 1 and Table 2), with global significant differences among the five groups compared (*p* < 0.0001 for the Kruskal–Wallis test). The differences were statistically significant for the pairwise comparison of AC21‐22 versus AC22‐23 and marginally significant for the comparison between AC19‐20 versus AC20‐21, and AC22‐23 versus AC23‐24. A significant correlation was found between AC and the scores obtained by the students (Kendall's tau *p* < 0.0001, *r* = 0.1318, and linear trend *R* ^2^ = 0.6241). When results were analyzed only for male students, results ranged between 5.40 ± 1.73 for AC23‐24 and 6.43 ± 1.33 for AC20‐21, with global differences among the five ACs, although only the comparison between AC21‐22 and AC22‐23 was marginally significant for the pairwise comparisons. For female students, the lowest results (5.07 to ±1.68) were obtained in AC22‐23, and the highest scores (6.28 ± 1.61), in AC19‐20. Global differences among all ACs were statistically significant, and the pairwise comparisons of AC21‐22 versus AC22‐23, AC19‐20 versus AC20‐21, and AC22‐23 versus AC23‐24 were only marginally significant. For both genders, a significant correlation between the AC and student performance was found (*p* < 0.0001 and *r* = 0.1763 for men and *p* < 0.0001 and *r* = 0.1299 for women).

**TABLE 2:** Performance of the students enrolled in each academic year (AC) expressed as final scores.

Scores	Final score		
	All	Males	Females
AC19‐20	6.29 ± 1.71	6.32 ± 1.93	6.28 ± 1.61
AC20‐21	5.81 ± 1.71	6.43 ± 1.33	5.77 ± 1.65
AC21‐22	6.02 ± 1.65	6.28 ± 1.76	5.89 ± 1.59
AC22‐23	5.17 ± 1.72	5.43 ± 1.79	5.07 ± 1.68
AC23‐24	5.53 ± 1.8	5.4 ± 1.73	5.59 ± 1.84
PRE	6.05 ± 1.73	6.38 ± 1.64	6.03 ± 1.65
INT	6.02 ± 1.65	6.28 ± 1.76	5.89 ± 1.59
POST	5.35 ± 1.77	5.42 ± 1.75	5.31 ± 1.77
AC19‐20 vs. AC20‐21	0.0020^M^	0.9038	0.0065^M^
AC20‐21 vs. AC21‐22	0.3097	0.7965	0.8213
AC21‐22 vs. AC22‐23	<0.0001*	0.0023^M^	<0.0001*
AC22‐23 vs. AC23‐24	0.0198^M^	0.7652	0.0040^M^
PRE vs. POST	<0.0001*	<0.0001*	<0.0001*
PRE vs. INT	0.6815	0.7788	0.2531
POST vs. INT	<0.0001*	0.0001*	0.0010^M^

*Note*: Results are shown as average ± standard deviations and correspond to the final score results obtained by the students included in each study group (from 0 to 10 points). Results are shown for the whole group of students (ALL), for male and female students, and for students enrolled in each AC and in the PRE, INT, and POST groups. The last rows show the *p* values for the statistical comparisons carried out in the present work. PRE: Students who received pre‐COVID‐19 pre‐university education; INT: Students who received a mixed intermediate pre‐university education system; POST: Students enrolled in post‐COVID‐19 pre‐university education. Statistically significant *p* values of <0.001 are highlighted with asterisks (*), whereas *p* values between 0.05 and 0.001 are labeled with “M”.

When PRE students were overall compared with POST students, we found that the average scores corresponding to the PRE group were significantly higher than those of the POST group (*p* < 0.0001). Interestingly, the scores of the PRE students were similar to those of the INT students, with non‐significant differences between the two groups (Figure 1 and Table 2). The correlation between students' scores and the type of study previously conducted (PRE or POST) was statistically significant (Kendall tau *p* < 0.0001, *r* = 0.1658, and linear trend *R* ^2^ = 0.7821), and the binary logistic regression analysis was statistically significant (*p* < 0.0001). Similar results were obtained when the results were analyzed for a specific gender, with significant differences between PRE and POST students, but not between PRE and INT students, and with a significant correlation between the scores and the type of study (Kendall tau *p* = 0.0002 and *r* = 0.1527 for men, and Kendall tau *p* < 0.0001 and *r* = 0.1671 for women).

### Analysis of correct, incorrect, and unanswered items

**FIGURE 2:**
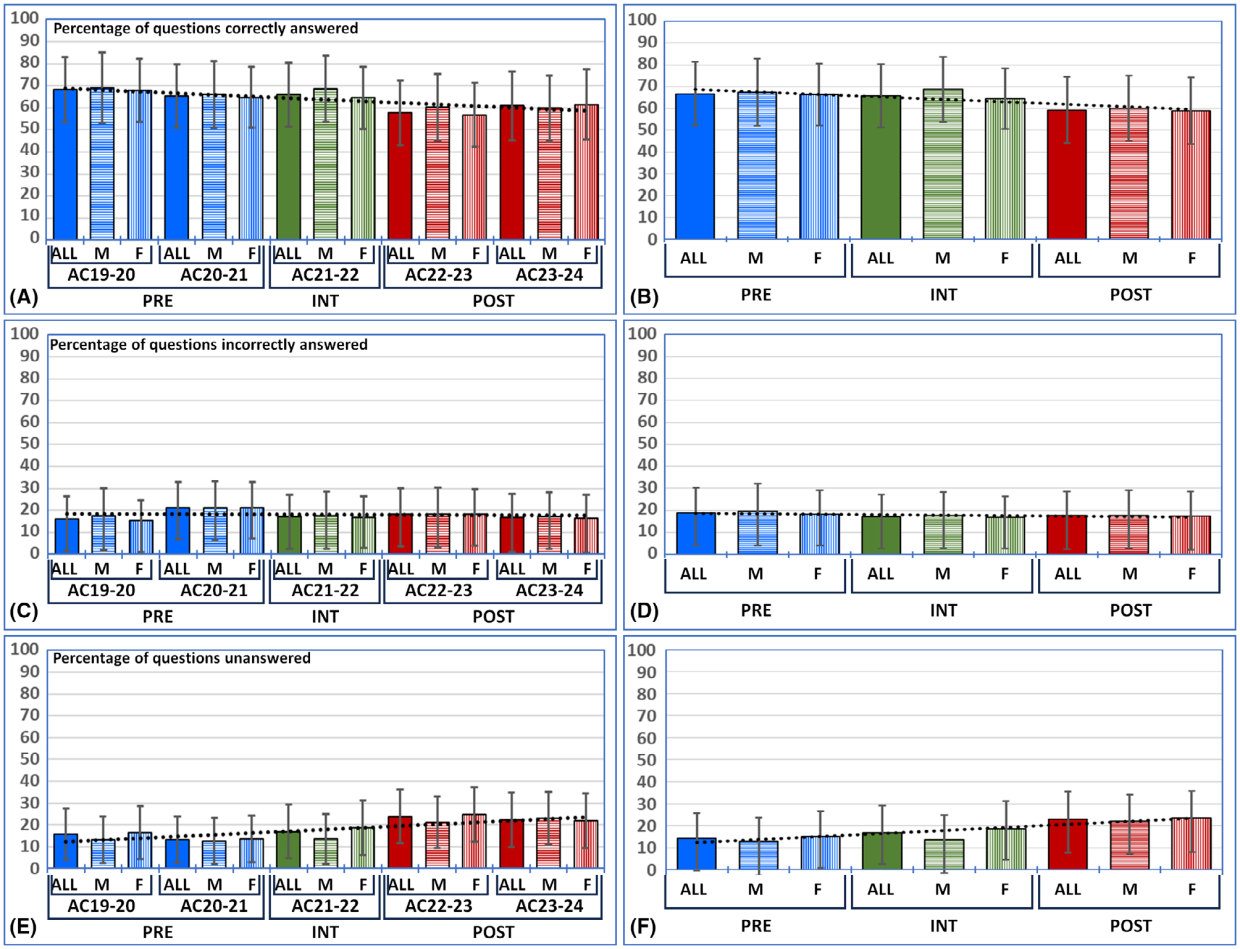
Percentage of questions answered correctly (panels A and B), incorrectly (panels C and D) and unanswered (panels E and F) by each student. Results are shown as average percentages for students enrolled in each academic year (panels A, C, and E) and the PRE, INT and POST groups (panels B, D, and F), for the whole group of students (ALL), and for male (M) and female (F) students only. Error bars correspond to standard deviations. PRE: Students who received pre‐COVID‐19 pre‐university education; INT: Students who received a mixed intermediate pre‐university education system; POST: Students enrolled in post‐COVID‐19 pre‐university education. The black dotted lines correspond to linear trends.

When the percentage of items answered correctly by the students was analyzed (Figure 2 and Table 3), we found significant overall differences among the five ACs compared (*p* < 0.0001 for the Kruskal–Wallis test), with significant differences for the pairwise comparison of AC21‐22 versus AC22‐23, AC19‐20 versus AC20‐21, and AC22‐23 versus AC23‐24 being marginally significant. Values obtained in the PRE group were significantly higher than those in the POST group but comparable to those in the INT group. A significant correlation was found between the AC and the percentage of correct answers (Kendall tau *p* < 0.0001 and *r* = 0.1523), and the binary logistic regression analysis was statistically significant (*p* < 0.0001). Similar results were found for only female students, with significant differences for the same pairwise comparisons and a significant correlation with the AC (Kendall tau *p* < 0.0001 and *r* = 0.1485). However, the analysis carried out for male students only revealed marginally significant pairwise differences for the comparison of AC21‐22 versus AC22‐23, and the correlation with the AC was not statistically significant (*p* = 0.6743).

**TABLE 3:** Performance of the students enrolled in each academic year (AC) shown as percentage of correct, incorrect and unanswered questions.

Scores	Correct answers			Incorrect answers			Unanswered		
	All	Males	Females	All	Males	Females	All	Males	Females
AC19‐20	68.31 ± 14.81	69.06 ± 16	67.98 ± 14.31	16.09 ± 10.29	17.65 ± 12.55	15.41 ± 9.1	15.61 ± 11.79	13.29 ± 10.65	16.6 ± 12.14
AC20‐21	65.23 ± 14.28	66.02 ± 15.12	64.8 ± 13.84	21.25 ± 11.8	21.33 ± 12.13	21.21 ± 11.66	13.16 ± 10.67	12.65 ± 10.68	13.43 ± 10.69
AC21‐22	65.95 ± 14.48	68.73 ± 15	64.47 ± 14.02	17.12 ± 10.02	17.69 ± 10.73	16.81 ± 9.64	16.93 ± 12.29	13.58 ± 11.33	18.72 ± 12.44
AC22‐23	57.85 ± 14.67	60.43 ± 15.03	56.84 ± 14.44	18.41 ± 11.48	18.36 ± 12.23	18.43 ± 11.21	23.75 ± 12.32	21.21 ± 11.86	24.73 ± 12.39
AC23‐24	60.88 ± 15.6	59.81 ± 14.9	61.42 ± 15.97	16.81 ± 10.64	17.29 ± 10.81	16.57 ± 10.58	22.31 ± 12.38	22.9 ± 12.15	22.01 ± 12.53
PRE	66.71 ± 14.61	67.37 ± 15.55	66.39 ± 14.15	18.77 ± 11.38	19.7 ± 12.42	18.32 ± 10.84	14.33 ± 11.28	12.93 ± 10.64	15.01 ± 11.53
INT	65.95 ± 14.48	68.73 ± 15	64.47 ± 14.02	17.12 ± 10.02	17.69 ± 10.73	16.81 ± 9.64	16.93 ± 12.29	13.58 ± 11.33	18.72 ± 12.44
POST	59.33 ± 15.19	60.1 ± 14.91	59 ± 15.33	17.63 ± 11.09	17.79 ± 11.46	17.55 ± 10.94	23.04 ± 12.36	22.11 ± 12	23.45 ± 12.51
AC19‐20 vs. AC20‐21	0.0140^M^	0.9651	0.0394^M^	<0.0001*	0.2086	<0.0001*	0.0198^M^	0.3838	0.0124^M^
AC20‐21 vs. AC21‐22	0.7807	0.7577	0.5638	<0.0001*	0.4388	0.0001*	0.0004*	0.3758	<0.0001*
AC21‐22 vs. AC22‐23	<0.0001*	0.0009*	<0.0001*	0.1832	0.8202	0.1340	<0.0001*	0.0001*	<0.0001*
AC22‐23 vs. AC23‐24	0.0204^M^	0.7739	0.0044^M^	0.0689	0.7396	0.0409^M^	0.1402	0.5341	0.0367^M^
PRE vs. POST	<0.0001*	0.0017^M^	<0.0001*	0.2257	0.0011^M^	0.6123	<0.0001*	<0.0001*	<0.0001*
PRE vs. INT	0.3641	<0.0001*	0.0910	0.0792	0.0024^M^	0.1405	0.0068^M^	0.0351^M^	0.0011^M^
POST vs. INT	<0.0001*	<0.0001*	0.0006*	0.6305	0.9776	0.5253	<0.0001*	<0.0001*	0.0001*

*Note*: Results are shown as average ± standard deviations and correspond to the percentage of items answered correctly in each study group (Correct answers), the percentage of items answered incorrectly in each group of study (Incorrect answers), and the percentage of unanswered items in each group of study (Unanswered), from 0 to 10 points. Results are shown for the whole group of students (ALL), for male and female students, and for students enrolled in each AC and the PRE, INT, and POST groups. The last rows show the *p* values for the statistical comparisons carried out in the present work. PRE: Students who received pre‐COVID‐19 pre‐university education; INT: Students who received a mixed intermediate pre‐university education system; POST: Students enrolled in post‐COVID‐19 pre‐university education. Statistically significant *p* values of <0.001 are highlighted with asterisks (*), whereas *p* values between 0.05 and 0.001 are labeled with “M”.

For the percentage of items answered incorrectly (Figure 2 and Table 3), overall differences were found among the five ACs compared (*p* < 0.0001 for the Kruskal–Wallis test), with pairwise comparisons revealing significant differences only for AC19‐20 versus AC20‐21 and AC20‐21 versus AC21‐22. However, the differences were not significant when comparing the PRE, POST, and INT groups. The correlation of the percentage of incorrect answers with the AC was not statistically significant (*p* = 0.6257), and the same trend was found in the binary logistic regression (*p* = 0.1070). A similar pattern was observed for the group of female students, with significant overall differences among the five ACs. Pairwise comparisons were significant for AC19‐20 versus AC20‐21 and AC20‐21 versus AC21‐22, with AC22‐23 versus AC23‐24 being marginally significant, although the correlation with the AC was not statistically significant. For men, however, we found that overall comparisons between the five ACs were non‐significant, as were pairwise comparisons. The percentage of incorrect answers was only marginally higher in the POST group than in the PRE group, and the correlation between this percentage and the AC was also marginally significant (Kendall tau *p* = 0.0203 and *r* = 0.0879).

Regarding unanswered questions (Figure 2 and Table 3), our analysis showed significant overall differences among all the ACs (*p* < 0.0001 for the Kruskal–Wallis test) and pairwise differences for AC20‐21 versus AC21‐22 and AC21‐22 versus AC22‐23, with AC19‐20 versus AC20‐21 being marginally significant. The comparisons of the PRE versus POST and POST versus INT groups were statistically significant, whereas that of PRE versus INT was marginally significant. The lowest percentages corresponded to the PRE group and the highest to the POST group. A significant correlation was found between the percentage of unanswered questions and the AC (Kendall tau *p* < 0.0001 and *r* = 0.2086), with a significant *p*‐value in the binary logistic regression analysis (*p* < 0 0.0001). A similar trend was observed in the female group, although the comparison between AC22‐23 and AC23‐24 was marginally significant. For all students, a significant correlation with the AC was found (Kendall tau *p* < 0.0001 and *r* = 0.1923). For male students, significant overall differences among all the ACs were found (*p* < 0.0001 for the Kruskal–Wallis test), but pairwise comparisons were only significant for AC21‐22 versus AC22‐23, and not for the rest of the comparisons. For women and all students, differences were found between the PRE and POST and the POST and INT groups.

## DISCUSSION

In the present work, we conducted a performance analysis of the results obtained by a group of students of the medical school who received pre‐COVID‐19 education before enrolling in medical school, and we compared these results with those of students who received post‐COVID‐19 high school education, revealing several crucial differences between these groups. An important issue is whether the type of examination and the difficulty of the exams used to evaluate the students' performance during five ACs were similar and whether teaching was conducted by the same teachers. To answer this question, we used a multifactorial consistency analysis approach including three variables (*α*, DI, and PB) to increment the potency of the analysis.^16^ The results demonstrated that all the exams had high reliability and that their difficulty was similar. In this regard, it is important to note that the evaluation test applied to the students included in the present study consisted of a multiple‐choice question exam whose characteristics, types of questions, and difficulty have remained unmodified for the last five ACs. Therefore, it was expected that the *α*, DI, and PB values could offer similar results for the five exams included in the present study, and the differences among ACs should not be attributed to the characteristics of the evaluation exams.

When students' performance was evaluated, we found that the scores of students enrolled in a subject taught in the first semester of the medical curriculum differed significantly during the last five ACs. Specifically, the results significantly decreased for students who received POST instruction before enrolling in medical school, and this trend was consistent for both male and female students. These results are in line with previous reports suggesting that POST teaching adaptations might have reduced medical students' knowledge and preparation for becoming future medical doctors.^19^, ^20^, ^21^ However, a recent study showed that the changes implemented during the pandemic did not affect specific skills, such as the ability of dental students to interpret craniofacial radiologic images.^22^ Interestingly, a recent randomized trial was designed to determine the outcomes of postgraduate health and medical students subjected to online and on‐site teaching.^23^ Although the results are not yet available, this trial is expected to shed light on the effects of non‐presential teaching on medical students.

On the other hand, we aimed to determine specific factors that could be associated with the different performances of PRE and POST students. For this purpose, we analyzed the percentage of items that the students answered correctly, incorrectly, or left unanswered and found significant differences among the groups. Strikingly, our results revealed a significant trend toward a lower percentage of questions correctly answered over time, with significant differences between PRE and POST students, and a correlation with the AC, with few differences between genders. In contrast, the percentage of items that were incorrectly answered remained constant and varied very little among ACs, suggesting that PRE and POST students answered incorrectly in approximately the same proportion in both periods. Finally, the analysis of unanswered questions showed a significant correlation with the AC and a significant increase in the percentage of blank answers among POST students, with few differences between men and women. These results suggest that the performance differences between PRE and POST students could be related to the higher tendency of PRE students to answer doubted items, whereas POST students tended to leave more questions unanswered; however, the percentage of failure was approximately the same across all student groups.

An important factor influencing the teaching‐learning process is the method used during learning and the teaching tools and resources implemented by teachers.^24^ The relevance of the teaching modifications adopted during the pandemic, including the adoption of e‐learning and digital resources in medical education,^25^ remains to be determined, but these changes likely influenced the results of the present study. Although we mainly focused on the analysis of student performance, we cannot exclude the possibility that the differences found between the PRE and POST groups could be related to different teaching tools used in teaching Cell Biology and Principles of Human Inheritance in these groups because of pandemic restrictions. However, as non‐presential teaching with classes given through the website was only used for one of the PRE years (AC20‐21), we might hypothesize that the effect of virtual teaching on students' performance could not be critical. Future studies should determine the effects of teaching tools on the capability of medical students to acquire skills and knowledge related to this subject in the medical curriculum.

Interestingly, our study showed that students who received a mixed model of pre‐university teaching (INT group) obtained similar results to those of the PRE group, suggesting that pre‐university teaching changes affected students' performance only when the entire high school period was affected (POST group). As the PRE group received presential onsite teaching at the medical school, 1 year of presential teaching, and 1 year of virtual teaching at the pre‐university level, it is probable that the effects of non‐presential learning were not strong enough to alter students' performance unless the entire period of pre‐university learning was affected by this type of teaching.

Multiple factors can influence the likelihood of students leaving their questions unanswered on examination tests. In research questionnaires, participants with lower previous formation and literacy failed to answer questionnaire items with a higher probability than participants with higher formation.^26^ In medical students, the lack of certainty is the main factor promoting avoidance of decision‐making, and students with lower levels of confidence and higher aversion to ambiguity tend to leave more questions unanswered and obtain lower final scores.^27^ This phenomenon is relevant because confidence and confrontation with uncertainty are important requirements of medical practice, where doctors must commonly face the uncertainty of diagnosis, therapy, and patient outcomes.^28^ The fact that POST students may have lower confidence in their own knowledge and capabilities is worrisome and requires specific training programs to reinforce their own potential. It has been suggested that this specific aspect of medical education is essential to the medical curriculum.^29^ The reasons POST students may have lower confidence and a higher degree of uncertainty remain unknown and should be determined in future studies. However, we might hypothesize that POST high school education, in which students had to confront the uncertainty of the school system, examination methods, evolution of the disease, and many other sources of anxiety and ambiguity, could have critically impacted the way students faced their current duties at the medical school. This is in agreement with several reports suggesting that post‐COVID‐19, university students have high degrees of uncertainty and that interventions targeted at controlling intolerance to uncertainty should be implemented.^30^ In addition, male and female students may differ in their tendency to answer uncertain questions, and women may have a higher tendency to leave uncertain questions unanswered.^31^ Although the results of our study suggest that gender differences are not significant, future studies should specifically assess this issue.

The present study has several limitations. First, this was a retrospective study in which students were analyzed at different time points (AC). Despite the same programs and teachers, it is clear that differences may exist over different academic years. Although our analyses revealed that the exams were similar across years, specific differences might have influenced the students' performance. Moreover, the different teaching tools and strategies used in each AC could also have influenced the results, especially for students subjected to non‐presential teaching for the entire academic year.

In summary, the present work demonstrates that POST students tend to show lower performance in the exams conducted at the beginning of their medical school curriculum and that this reduction is mainly related to a lower percentage of answered questions that could be associated with higher degrees of uncertainty. Although multiple factors could affect the results and percentage of students' answers, these findings shed light on the potential effects of the pandemic on students accessing medical schools. Future formative programs may be implemented for POST medical students to reinforce their skills and capabilities.

## AUTHOR CONTRIBUTIONS

**José Manuel García:** Conceptualization; investigation; supervision; writing – original draft. **David Sanchez‐Porras:** Conceptualization; investigation; supervision; writing ‐ original draft. **Miguel Etayo‐Escanilla:** Formal analysis. **Paula Ávila‐Fernández:** Formal analysis. **Olimpia Ortiz‐Arrabal:** Formal analysis. **Miguel‐Ángel Martín‐Piedra:** Formal analysis. **Fernando Campos:** Formal analysis. **Óscar‐Darío García‐García:** Data curation; formal analysis; investigation; writing – review and editing. **Jesús Chato‐Astrain:** Data curation; formal analysis; investigation; writing – review and editing. **Miguel Alaminos:** Conceptualization; investigation; supervision; writing – original draft.

## FUNDING INFORMATION

This study was supported by Tissue Engineering Group of the University of Granada (CTS‐115).

## CONFLICT OF INTEREST STATEMENT

The authors declare that they have no competing interests.

## ETHICS APPROVAL AND CONSENT TO PARTICIPATE

This study was approved by the Ethics and Research Committee of the University of Granada (ref. 4030/CEIH/2024).

## Data Availability

Quantitative data can be accessed at the open‐access European Research Data Repository Zenodo (https://zenodo.org/records/10687862).
